# Disseminated cryptococcosis with recurrent multiple abscesses in an immunocompetent patient: a case report and literature review

**DOI:** 10.1186/s12879-017-2459-9

**Published:** 2017-05-30

**Authors:** Qiaoling Ruan, Yimin Zhu, Shu Chen, Liping Zhu, Shu Zhang, Wenhong Zhang

**Affiliations:** Department of infectious disease, Huashan Hospital of Fudan University, Shanghai, China

**Keywords:** Cryptococcosis, Immunocompetent, Abscess

## Abstract

**Background:**

*Cryptococcus neoformans* is frequently present as an opportunistic pathogen mainly affecting immunocompromised populations. Disseminated *C. neoformans* infection in immunocompetent population is rare and usually involves lung and central nerve system. Cryptococcus from biologic samples can easily grow on routine fungal and bacterial culture media. Besides, cryptococcal latex agglutination test has been established as a reliable diagnostic tool with overall sensitivities of 93–100%.

**Case presentation:**

We report a rare disseminated cryptococcosis case which presented with chronic recurrent multiple abscess in an immunocompetent male involving skin, lung, spine and iliac fossa without evidence of central nerve system involving. The results of serum cryptococcal latex agglutination tests and standard microbial cultures were negative. The patient underwent empirical anti-bacterial and anti-tuberculosis therapy which turned out to be effectless. Finally, bedside inoculation of the pus was carried out and revealed *Cryptococcus neoformans*, which was confirmed by polymerase chain reaction. After the administration of anti-fungal drugs including liposomal amphotericin B, the patient recovered from fever and paraplegia.

**Conclusions:**

This case reveals an uncommon pattern of disseminated *C. neoformans* infection in immunocompetent population presented with chronic multiple abscess and without central nerve system involving. Negative routine microbial cultures may not necessarily rule out cryptococcosis, especially in early stage. Besides, cryptococcal latex agglutination test does have a chance of false negative, which might be related with “capsule-deficiency”. Moreover, this phenomenon could be related with low-grade virulence and relative long illness duration.

## Background

*Cryptococcus neoformans* is generally considered as an opportunistic pathogen because of its tendency to cause infection in immunocompromised individuals, particularly individuals with HIV infection. The lungs are predominantly the primary locus of infection, with extra-pulmonary dissemination through hematogenous route affecting the meninges and, less commonly, the skin, bones, prostate, and other organs. The present report describes a rare case of disseminated cryptococcosis which presented with recurrent multiple abscesses involving skin, lung, spine and iliac fossa in a patient with no detectable immune deficiencies and negative results of serum cryptococcal latex agglutination tests (SCLATs) and routine microbial cultures.

## Case presentation

**Fig. 1:**
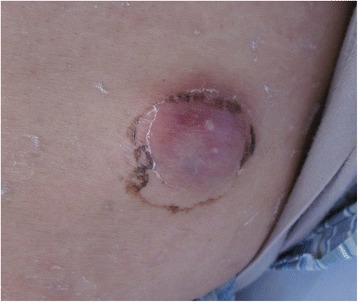
The lump on the back: There was a bean-size lump with slight tenderness on the left side of the lower back

**Fig. 2:**
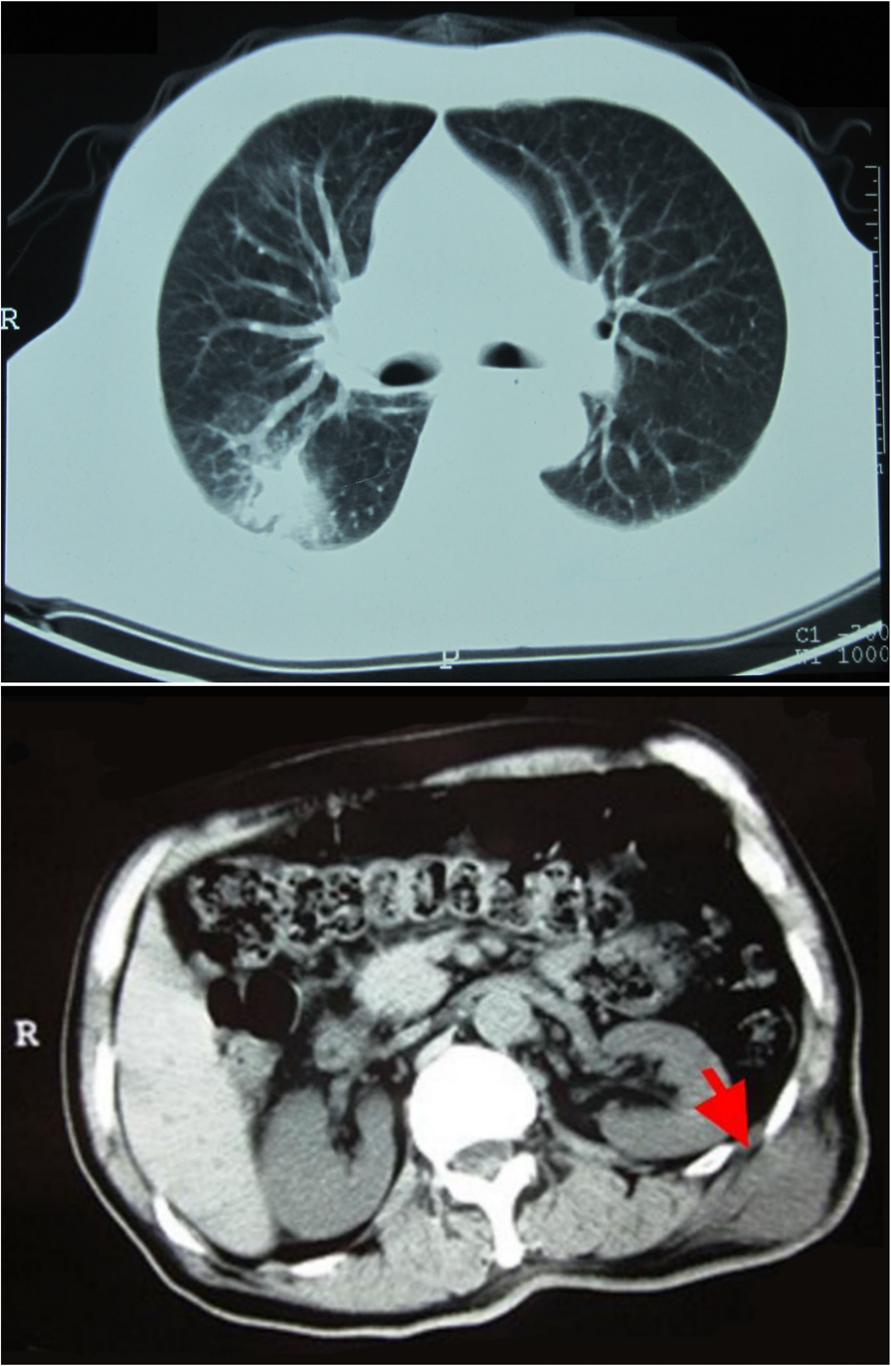
Abscess in lung and fascia: Chest CT scan revealed pulmonary abscess formation (*above*) and an abscess inside the fascia (*below*)

**Fig. 3:**
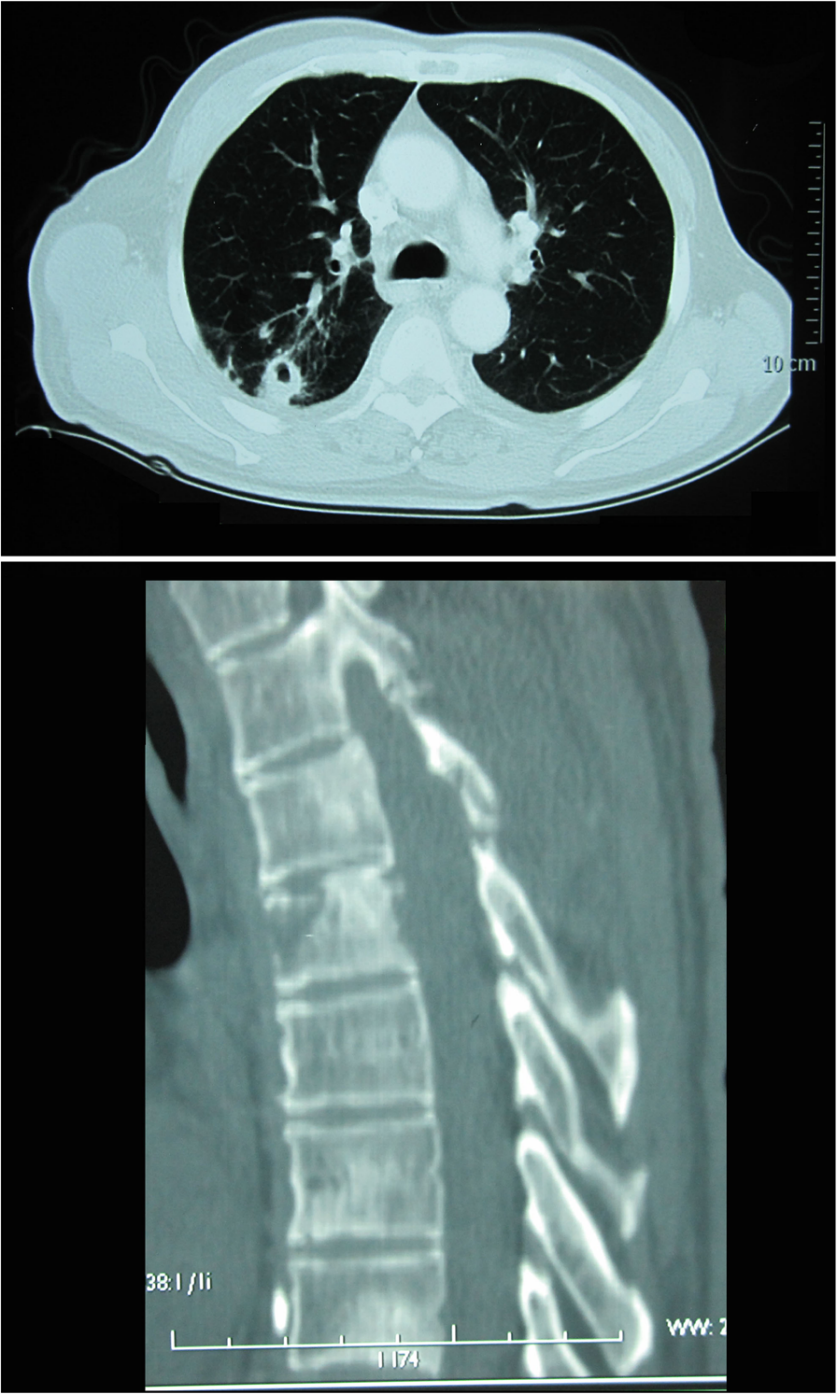
CT scan revealed progressing infection: Chest CT scan revealed the unresolved pulmonary abscess (*above*) compared with 2 months before (Fig. 2). Moreover, there were additional multiple destructions of vertebral bodies, especially T5 in the figure below

A 68-year-old man presented with a 7-month history of progressive multiple abscesses, followed by fever, lower extremity weakness and urinary retention. Seven months before, he occasionally found a bean-size lump with slight tenderness on the left side of the lower back. One month later, the lump increased to the size of an egg (Fig. 1). Computed tomography (CT, for short) scan revealed an abscess inside the fascia. Chest CT scan showed pulmonary abscess formation (Fig. 2). Repeated cultures of blood and percutaneous aspiration were constantly negative. Abscess incision and drainage were performed and empirical antibiotic treatment was given. Three months later, chest CT revealed the unresolved pulmonary abscess and additional multiple destructions of vertebral bodies, especially T5 and T11 (Fig. 3). Empirical anti-tuberculosis drugs were administrated for 2 months. Instead of improvement, the patient became febrile to 38.4 °C, and developed weakness and numbness in both legs and urinary retention. The patient reported no significant past medical history, and he denied any exposure to bird droppings.

On examination, physician identified a skin lesion with draining sinus on the left side of the lower back. He had grade 0 muscle strength of both legs and slightly increased muscle tone. There was slight tenderness on the T4 and T5 vertebrae bodies, with hypoesthesia below T4 level. Laboratory testing revealed a leucocyte count of 16.55 × 10^3^ cells/μL, a C-reactive protein concentration of 128 mg/L, an erythrocyte sediment rate of 120 mm/h and a negative result of HIV serology test. CD4^+^ T cell count was normal and the levels of serum globulins including IgG, IgA, IgM and total IgE were within normal range. T-SPOT. *TB* was negative. The (1, 3)-β-D-glucan test (or G test) and repeated SCLATs were all negative. Other laboratory results were unremarkable.

**Fig. 4:**
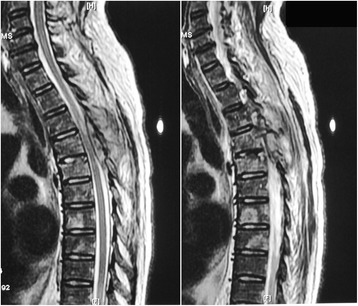
Bone destructions and compressed spine: These two sagittal views of spinal MRI T2-weighted sequence revealed multiple bone destructions of thoracic vertebrae, with spine compressed at T4 and T5 level

Spinal magnetic resonance imaging (MRI, for short) and subsequent CT revealed multiple bone destruction of thoracic and lumber vertebrae and ribs, with spine compressed at T4 and T5 level (Fig. 4). Surgical excision of paravertebral abscess at T4-T5 level was performed and pathological examination of the excisional biopsy specimen found nothing but inflammatory changes. Specimen cultures were negative for bacteria, tuberculosis and fungi. Anti-mycobacterial treatment was ceased and the antibiotics changed to intravenous sulfamethoxazole-trimethoprim compound and oral doxycycline. Gradually, his temperature returned to normal and abscesses regressed. The patient was discharged on oral maintenance therapy.

**Fig. 5:**
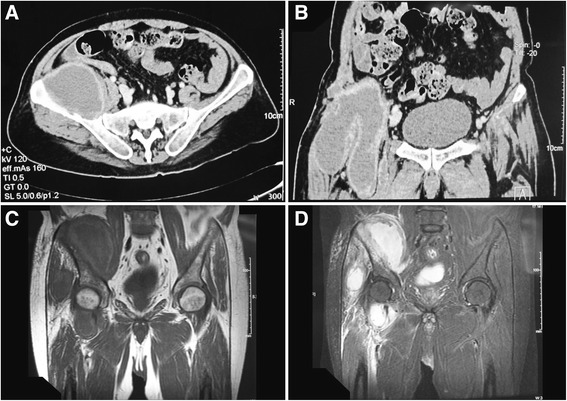
Contrasted CT scan and non-contrasted MRI scan of pelvic cavity: **a** Axial view of CT scan. **b** Coronal view of CT scan. **c** Coronal view of MRI T1-weighted fast spin echo sequence. **d** Coronal view of MRI short TI inversion recovery sequence. Both CT and MRI scan revealed a large right iliac abscess

**Fig. 6:**
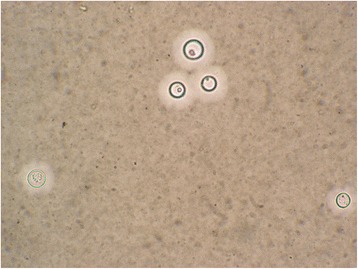
India ink staining of the pus culture: The cryptococcus can be found in the pus culture after the India ink staining

**Fig. 7:**
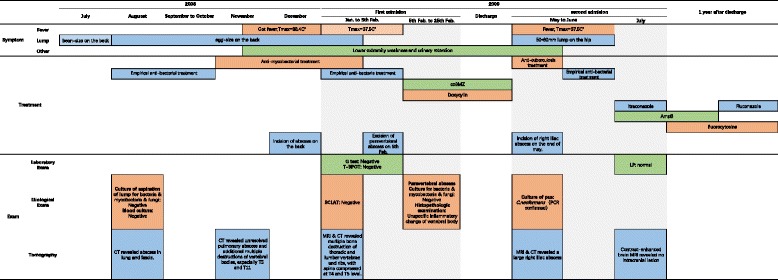
Gantt chart: The Gantt chart illustrates the course of the patient’s disease

Two months later, the patient presented to hospital again with a complaint of fever and a 50 × 60 mm new-onset lump with slight tenderness in the right groin. CT and MRI revealed a large right iliac abscess (Fig. 5). Repeated cultures were negative and laboratory tests revealed no specific findings. The abscess increased in size gradually. Surgical drainage yielded 700 ml of coffee ground pus. Bedside inoculation of the pus was carried out and the result of culture revealed *Cryptococcus neoformans*, which was confirmed by polymerase chain reaction (PCR, for short) (Fig. 6). The contrasted brain MRI found no evidence of central nervous system involving and so did the lumbar puncture. Liposomal amphotericin B and itraconazole were given. The fevers resolved when the accumulated dose of amphotericin B reached 2.5 g, and then itraconazole was replaced by fluorocytosine. We withdrew liposomal amphotericin B when its accumulated dose reached 3.0 g. The patient discharged with 4 months fluorocytosine plus fluconazole and regular follow-up. At 18-month follow-up, the patient was afebrile, with no new-onset abscess, and he could walk slowly. A Gantt chart was used to summarize the presentation and management of the patients (Fig. 7).

## Discussion and conclusions

Cryptococcosis is an opportunistic infectious disease caused by encapsulated yeasts in the genus *Cryptococcus.* Two species, *C. neoformans* and *C. gattii*, commonly cause disease in humans. *C. neoformans* can cause cryptococcosis in both immunocompetent and immunocompromised patients, while *C. gattii* usually infects apparently immunocompetent hosts. However, the percentage of *C. gattii* infection causing disease in apparently normal hosts is significantly higher than for *C. neoformans* [1]*.* After a primary infection in the lungs, the disease can either localized or disseminate through blood to various organs, depending on patients’ immune status. Disseminated cryptococcosis in immunocompetent population is rare and usually involves the central nerve system [2].

In the present case report, the patient was otherwise healthy and presented with progressive multiple abscesses caused by *C. neoformans* without cryptococcal meningitis, which was quite rare and was easily been misdiagnosed. To our knowledge, only five cases with extra-pulmonary and extra-cranial cryptococcal abscess has been reported in immunocompetent patients up to now (Table 1), but none of them was presented with such recurrent multiple soft-tissue abscesses for more than half a year like this case [3–7].

**Table 1:** Case reports on extra-pulmonary and extra-cranial cryptococcal abscess

Case	Presentation		Diagnosis	Management
	Pattern	Comorbidities		
Al-Tawfiq JA et al. 2007 [3]	Vertebral abscess, Lung lesion?^a^	Axillary lymph nodes TB	Pus culture: Positive SCLAT^b^: Negative	Surgical excision of vertebral abscess Fluconazole
Singh R et al. 2010 [4]	Psoas abscess sternum & vertebral involved	Pulmonary TB Abortion Varicella infection	FNAC^c^: Positive SCLAT: Positive	Amphotericin B
Gaskill T et al. 2010 [5]	Soft tissue abscess, Lung & Mediastinal lymph nodes involved	Remote history of depression	Biopsy: Positive SCLAT: Positive Tissue culture: Positive	Surgical excision of thigh abscess Fluconazole
Suchitha S et al. 2012 [6]	Soft tissue & cerebral abscess, Lung involved	Diabetes	FNAC: Positive Sputum culture: Positive	No surgery Fluconazole & Amphotericin B
Lenz D et al. 2015 [7]	Subcutaneous abscess	None	Tissue culture: Positive SCLAT: Negative	Surgical incision without anti-fungal drug

^a^The tissue cultures found only Cryptococcus in vertebral abscess and *M. tuberculosis* in axillary lymph nodes. The patient had no productive cough and thus sputum cultures were not obtained. The pathogen of pulmonary lesion remains unclear

^b^SCLAT: Serum Cryptococcus Latex Agglutination Test

^c^FNAC: Fine Needle Aspiration Cytology

At onset, the patient presented with cutaneous and pulmonary infection. Most cutaneous infection occurs as a sign of disseminated cryptococcal infection, which can be seen in 10–15% of the cases [8]. Cryptococcosis can present with a variety of skin manifestations including acneiform lesions, abscesses, vesicles, purpura, nodules, ulcers, granulomas, pustules, draining sinuses and cellulitis [9, 10]. The examination of involved skin tissue including biopsy or tissue culture can be helpful for diagnosis [3]. However, in this case, the skin lesion was non-specific and cultured negative. In high tuberculosis burden area, disseminated tuberculosis should be ruled out carefully especially when cryptococcusis is complicated with TB.

Confirmative diagnosis of disseminated cryptococcosis usually relies on positive culture or pathological results. In other similar cases, misdiagnosis is common and the examination of samples from invasive operations plays a vital role in accurately diagnosis. *Cryptococcus* from biologic samples can easily grow on routine fungal and bacterial culture media. However, our patient was negative for repeated cultures, which might indicate the insensitivity of culture at the early stage of infection. In additional, less fungaemia is seen in immunocompetent patients compared with immunocompromised ones [2]. CLAT detects the capsular polysaccharide antigens of *C. neoformans*, which has been established as a reliable diagnostic tool with overall sensitivities of 93–100% [11]. However, our patient had consistently negative results. In other similar cases, CLAT can also be negative [3, 7].

The capsule is composed of glucuronoxylomannan and galactoxylomannan polysaccharides. It is considered the one of the classical virulence factor for *C. neoformans,* and mutants without capsules are avirulent [12, 13]. Several *C. neoformans* genes in capsular synthesis and formation have already been identified, and site-directed gene mutants result in hypocapsular or acapsular strains. Capsule-deficient mutants are less virulent than the parental strains [14–17]. Considering that the patients had relatively long illness course, we inferred that he was infected with a strain of low-grade virulence which maybe hypocapsular. It could also explain the false-negative CLAT result, which has a high sensitivity. However, this is only speculation without available *C. neoformans* gene mutant detection.

## Abbreviations

CLAT: Cryptococcal latex agglutination tests
CT: Computed tomography
FNAC: Fine needle aspiration cytology
LP: Lumbar puncture
MRI: Magnetic resonance imaging
PCR: Polymerase chain reaction
SCLAT: Serum cryptococcal latex agglutination tests

## References

[1] Kwon-Chung KJ, Fraser JA, Doering TL, Wang ZA, Janbon G, Idnurm A, Bahn YS (2014). *Cryptococcus neoformans* and Cryptococcus gattii, the etiologic agents of Cryptococcosis. Cold Spring Harb Perspect Med.

[2] Lui G, Lee N, Ip M, Choi KW, Tso YK, Lam E, Chau S, Lai R, Cockram CS (2006). Cryptococcosis in apparently immunocompetent patients. QJM.

[3] Al-Tawfiq JA, Ghandour J (2007). *Cryptococcus neoformans* Abscess and osteomyelitis in an immunocompetent patient with tuberculous lymphadenitis. Infection.

[4] Singh R, Xess I (2010). Multiple osseous involvements in a case of disseminated cryptococcosis. Indian J Orthop.

[5] Gaskill T, Payne D, Brigman B (2010). Cryptococcal abscess imitating a soft-tissue sarcoma in an immunocompetent host: a case report. J Bone Joint Surg Am.

[6] Suchitha S, Sheeladevi CS, Sunila R, Manjunath GV (2012). Disseminated cryptococcosis in an immunocompetent patient: a case report. Case Rep Pathol.

[7] Lenz D, Held J, Goerke S, Wagner D, Tintelnot K, Henneke P, Hufnagel M (2015). Primary cutaneous cryptococcosis in an eight-year-old immunocompetent child: how to treat?. Klin Padiatr.

[8] Anderson DJ, Schmidt C, Goodman J, Pomeroy C (1992). Cryptococcal disease presenting as Cellulitis. Clin Infect Dis.

[9] Pema K, Diaz J, Guerra LG, Nabhan D, Verghese A (1994). Disseminated cutaneous cryptococcosis. Comparison of clinical manifestations in the pre-AIDS and AIDS eras. Arch Intern Med.

[10] Dimino-Emme L, Gurevitch AW (1995). Cutaneous manifestations of disseminated cryptococcosis. J Am Acad Dermatol.

[11] Tanner DC, Weinstein MP, Fedorciw B, Joho KL, Thorpe JJ, Reller LB (1994). Comparison of commercial kits for detection of Cryptococcal antigen. J Clin Microbiol.

[12] Perfect JR (2005). *Cryptococcus neoformans*: a sugar-coated killer with designer genes. FEMS Immunol Med Microbiol.

[13] Kronstad JW, Attarian R, Cadieux B, Choi J, D'Souza CA, Griffiths EJ, Geddes JM, Hu G, Jung WH, Kretschmer M (2011). Expanding fungal pathogenesis: Cryptococcus breaks out of the opportunistic box. Nat Rev Microbiol.

[14] Chang YC, Kwon-Chung KJ (1999). Isolation, characterization, and localization of a capsule-associated gene, CAP10, of *Cryptococcus neoformans*. J Bacteriol.

[15] Chang YC, Kwon-Chung KJ (1998). Isolation of the third capsule-associated gene, CAP60, required for virulence in *Cryptococcus neoformans*. Infect Immun.

[16] Chang YC, Kwon-Chung KJ (1994). Complementation of a capsule-deficient mutation of *Cryptococcus neoformans* restores its virulence. Mol Cell Biol.

[17] Alspaugh JA, Perfect JR, Heitman J (1997). *Cryptococcus neoformans* Mating and virulence are regulated by the G-protein alpha subunit GPA1 and cAMP. Genes Dev.

